# The influence of THC:CBD oromucosal spray on driving ability in patients with multiple sclerosis‐related spasticity

**DOI:** 10.1002/brb3.962

**Published:** 2018-04-06

**Authors:** Elisabeth G. Celius, Carlos Vila

**Affiliations:** ^1^ Department of Neurology Oslo University Hospital, and Institute of Health and Society University of Oslo Oslo Norway; ^2^ Almirall Neurology Global Medical Affairs Barcelona Spain

**Keywords:** driving ability, multiple sclerosis, oromucosal, Sativex, spasticity, tetrahydrocannabinol/cannabidiol

## Abstract

**Background:**

Driving ability is a key function for the majority of patients with multiple sclerosis (MS) to help maintain daily interactions. Both physical and cognitive disability, as well as treatments, may affect the ability to drive. Spasticity is a common symptom associated with MS, and it may affect driving performance either directly or via the medications used to treat it. In this article, we review the evidence relating the antispasticity medicine, Δ^9^‐tetrahydrocannabinol:cannabidiol (THC:CBD) oromucosal spray (Sativex^®^), and its potential impact on driving performance.

**Methods:**

Articles were identified by searching PubMed from 1/1/2000 to 30/6/2017 using a specified list of search terms. The articles identified using these search terms were augmented with relevant references from these papers and other articles known to the authors.

**Results:**

The results from THC:CBD oromucosal spray driving studies and real‐world registries did not show any evidence of an increase in motor vehicle accidents associated with THC:CBD oromucosal spray. The majority of patients reported an improvement in driving ability after starting THC:CBD oromucosal spray, and it was speculated that this may be related to reduced spasticity and/or better cognitive function. It should be noted that THC blood levels are significantly lower than the levels associated with recreational use of herbal cannabis.

**Conclusions:**

THC:CBD oromucosal spray was shown not to impair driving performance. However, periodic assessment of patients with MS driving ability is recommended, especially after relapses and changes in treatment. Blood THC measurements might be above authorized thresholds for some countries following administration of THC:CBD oromucosal spray, thus specific knowledge of each country's driving regulations and a medical certificate are recommended.

## 1 INTRODUCTION

Multiple sclerosis (MS) is a chronic, autoimmune disease caused by inflammation and neurodegeneration which is associated with a wide spectrum of central nervous system symptoms. The disease onset is usually in young adulthood, and common symptoms include fatigue, numbness, tingling, muscle spasticity, decreased cognition, and problems with balance and vision, as well as with bowel and bladder function. Commonly associated symptoms are physical and mental health comorbidities including back pain, arthritis, anxiety, sleep disorders, and depression (Kister et al., 2013; Pugliatti et al., 2006). MS has a significant negative impact on the patient's quality of life (QoL) compared with the general population (Lerdal, Celius, & Moum, 2003; McCabe & McKern, 2002; Murphy, Confavreux, & Haas, 1998).

The chronic, progressive course of MS is associated with physical (muscle paresis and spasticity), sensory (visual and auditory) symptoms, cognitive decline, and psychological impairments that may adversely affect functional independence in terms of activities associated with daily living, including interaction with family and friends, social activities, and ability to work (Freidel et al., 2015; Ryan et al., 2009). Mobility is an important component of functional independence, and the ability to drive a car is often integral to independence, social interactions, and activities of daily living such as access to work, family, shopping, and health care (Rapport, Hanks, & Bryer, 2006). Consequently, cessation of driving has the potential to markedly reduce the QoL in patients with MS.

Most research examining fitness to drive among persons with neurological impairment has focused on resumption of driving following an acute event, such as traumatic brain injuries (TBI) or stroke (Coleman et al., 2002; Schanke & Sundet, 2000). The course of MS is chronic/progressive albeit often with a number of acute episodes (relapses); therefore, factors related to TBI and stroke associated with fitness to drive may not be generalizable to the MS population. One study found that cognitive problems, awareness of deficit, and social influences on driving outcomes all played a key role in determining fitness to drive (Ryan et al., 2009). In this study, the majority of patients with MS continued to drive and had better neuropsychological functioning and awareness of deficits than nondrivers. Drivers unaware of their deficits perceived less need to develop compensatory behaviors, drove more, and had more driving‐related accidents. Importantly, the incidence of driving accidents was no different in patients with MS with normal cognition compared with healthy controls (Ryan et al., 2009).

In addition to disease‐related changes affecting driving ability, treatment‐related issues (mainly medication‐related) may also be important. As a group, patients with MS are treated with a multitude of drugs to manage their disease and its symptoms. These include disease‐modifying drugs, corticosteroids, and numerous agents to manage symptoms such as anxiety, depression, fatigue, pain, sleep disorders, bladder problems, and spasticity (Coclitu, Constantinescu, & Tanasescu, 2016; Vermersch, 2015).

The aim of the current review was to investigate factors pertaining to the use of the cannabinoid‐based medicine oromucosal Δ^9^‐tetrahydrocannabinol:cannabidiol [THC:CBD (Sativex^®^)] in patients with MS spasticity (MSS) to ascertain its impact on driving ability. Therefore, available data from observational studies and driving tests with THC:CBD oromucosal spray will be evaluated.

## 2 SEARCH STRATEGY

References for this review were identified by searching PubMed from 1 January 2000 to 30 June 2017. The terms “cannabinoids,” “Sativex,” and “tetrahydrocannabinol”; “cannabidiol,” “multiple sclerosis,” and “spasticity” were combined with the terms “driving ability,” “blood levels,” and “traffic accidents”. Articles identified using these search terms were augmented with concept‐related references known to the authors and others identified in the introduction/discussion sections from all identified papers.

## 3 MULTIPLE SCLEROSIS AND DRIVING

Patients with MS are more likely to be involved in motor vehicle accidents than people without MS (Knecht, 1977; Ling, 2002). In particular, patients with MS with cognitive impairment have a higher incidence of accidents compared with controls and patients with MS without cognitive impairment (Badenes et al., 2014; Schultheis, Garay, Millis, & Deluca, 2002). Various authors suggest that patients with MS should undergo frequent medical assessments and/or driving tests to retain their driving licenses given the progressive nature of the disease (Badenes et al., 2014; Knecht, 1977; Küst & Dettmers, 2014). During driving simulation testing, patients with relapsing–remitting MS had an increased number of accidents and concentration faults compared with healthy controls (Kotterba, Orth, Eren, Fangerau, & Sindern, 2003). Factors impacting driving skills included motor symptoms (weakness, coordination difficulties, paresis, and spasticity), visual and auditory disturbances, and cognitive deficits and depression (Marcotte et al., 2008). The most common cognitive deficits in MS are reduced attention, memory, and psychomotor speed, even early in the disease course (Landro, Sletvold, & Celius, 2000). A study examining the effects of cognitive dysfunction and spasticity on driving ability in patients with MS (*n* = 17) and healthy controls (*n* = 14) demonstrated that patients with MS exhibited great variability in driving simulation tests (Marcotte et al., 2008). In a multivariate model, cognitive function was the strongest predictor of difficulty maintaining lane position during a divided attention task and poorer response time to lead car speed changes, while spasticity was associated with reductions in accuracy of tracking the lead car movements and speed maintenance. One study showed that patients with MS who were unaware of their cognitive and physical deficits were less likely to engage in compensatory behaviors and were thus at greater risk of driving‐related accidents (Ryan et al., 2009).

The impact of MS treatments on driving ability has not been well studied. Searches for information regarding disease‐modifying drugs revealed no references assessing their effects on driving ability. Regarding symptomatic therapy, one small study with intrathecal baclofen for a mean duration of 22 months reported improvement in driving ability in three of four patients and deterioration in the fourth patient (Jagatsinh, 2009). Three studies with THC:CBD oromucosal spray are reported separately below.

## 4 DRUGS AND DRIVING: GENERAL LEGAL ISSUES AND LIMITS

According to World Health Organization (WHO) statistics, approximately 1.25 million people died from road traffic injuries in 2013, a 13% increase from 2000, and road traffic injuries are the main cause of death for people aged 15–29 years (World Health Organization, 2015). In an extensive review of driving under the influence of illicit substances of abuse, medications, or alcohol, 5%–25% of incidents involved drivers who tested positive for drugs (illicit substances of abuse or medications, excluding alcohol). It was noted that the drugs detected generally reflected usage patterns in the community (Kelly, Darke, & Ross, 2004), and polydrug usage was commonly detected. Interestingly, the authors noted that, between 1990 and 2004, alcohol as a cause of motor accidents decreased from 33% to 28%, whereas drug usage increased from 20% to 27%. Excluding alcohol, the prevalence of drugs considered to pose the greatest risk to traffic safety was cannabis (2%–32%), benzodiazepines (2%–15%), cocaine (4%–11%), amphetamines (2%–6%), and opioids (3%–5%).

The European Monitoring Centre for Drugs and Drug Addiction (EMCDDA) calculated odds ratios (OR) for crashes associated with injury based upon meta‐analyses and case–control studies (Verstraete, Legrand, Vandam, Hughes, & Griffiths, 2017). In order of decreasing risk OR (95% CI), drugs most frequently associated with accidents were as follows: alcohol 0–0.49 g/L (1.2; 0.8, 1.7), alcohol 0.5–0.79 g/L (3.6; 2.3, 5.7), alcohol 0.8–1.2 g/L (13.4; 8.2, 21.9), alcohol ≥1.2 g/L (62.8; 44.5, 88.6), amphetamines (6.2; 3.5, 11.1), cannabis (1.9; 1.4, 2.7), opioids (1.9; 1.5, 2.4), cocaine (1.7; 0.9, 3.0), benzodiazepines (1.6; 1.1, 2.3), antidepressants (1.3; 1.1, 1.7), and antihistamines (1.1; 1.0, 1.2).

In some countries, it is illegal to drive after the intake of approved medicines if they impair the ability to drive. In other countries, the governments specifically mention drugs such as amphetamines, benzodiazepines (clonazepam, diazepam, flunitrazepam, lorazepam, oxazepam, and temazepam), methadone, and opioids (morphine, codeine, tramadol, and fentanyl) (GOV.UK, 2017a,b). For most of the substances, which are viewed to be of higher risk for causing motor accidents, threshold blood limits have been defined. Levels exceeding these are considered to be a risk to road safety (Table 1) (GOV.UK, 2017a,b).

**Table 1:** Threshold limits (whole blood) for eight illegal drugs and eight high‐risk medicines (GOV.UK, 2017a,b)

Illegal drug (avoiding accidental exposure)	Threshold limit (μg/L)	Medicines (level constituting a risk)	Threshold limit (μg/L)
Benzoylecgonine	50	Clonazepam	50
Cocaine	10	Diazepam	550
Δ^9^‐tetrahydrocannabinol (THC) Cannabis	2	Flunitrazepam	300
Ketamine	20	Lorazepam	100
Lysergic acid diethylamide (LSD)	1	Methadone	500
Methylamphetamine	10	Morphine	80
Methylenedioxymethamphetamine (MDMA)	10	Oxazepam	300
6‐monoacetylmorphine (heroin)	5	Temazepam	1,000

In the case of amphetamine, a level is set (250 μg/L**)** which balances the risk between legal and illegal use.

Illicit drugs usually have zero tolerance. The UK applies this approach to eight of the most commonly used illicit drugs and sets threshold levels for these compounds specifically to rule out claims of accidental exposure (Table 1) (GOV.UK, 2017a,b). The problems arise for medications with active principles overlapping with drugs of abuse, such as opioid‐ or cannabinoid‐based medications, where patients follow the prescription and approved label recommendations but might still have, and be prosecuted for, a positive blood test.

## 5 CANNABIS AND CANNABINOIDS AND DRIVING

Worldwide herbal cannabis is the most widely used illicit substance (Substance Abuse and Mental Health Services Administration, 2011) and, in 2009, the United Nations Office on Drugs and Crime (UNODC) estimated that between 125 and 203 million individuals aged 15–64 years had used herbal cannabis at least once in the previous year, basically for recreational use (UNODC, 2011). In the United States, a 2007 National Roadside Survey reported cannabis as the most common illicit drug in drivers, with 8.6% of nighttime drivers tested positive for THC (Lacey et al., 2009). In a repeat survey in 2013/2014, the rate had increased to 12% (Berning, Compton, & Wochinger, 2017). These US figures are mirrored by many other countries around the world (Asbridge, Hayden, & Cartwright, 2012; European Monitoring Centre for Drugs and Drug Addiction, 2008; Neale, Mckeganey, Hay, & Oliver, 2000). In North America, a survey showed that 64% of patients with MS had tried cannabis before MS was diagnosed, 26% had used it as an MS treatment, and 16% were currently using cannabis (Cofield et al., 2017). Of note are the findings of an international working group that reported that acute herbal cannabis usage is associated with a significantly elevated risk of motor vehicle accidents (Asbridge et al., 2012; Rogeberg & Elvik, 2016). There are three main approaches for assessing whether a driver can drive under the influence of herbal cannabis:

1. Impairment‐based (effect‐based): assessment of the driver to perform certain tasks (the disadvantage is that there is no universally accepted methodology);
2. “Per se” laws which set a legal limit for the level of THC or its metabolites in the body (blood and saliva);
3. Zero tolerance a version of “per se”, but the legal limit is set at zero.

Due to the difficulties of assessing the impairment, and the limitations with respect to zero tolerance legislation, the main focus by most national traffic authorities has been to specify legal limits for THC (Wong, Brady, & Li, 2014). Table 2 shows current legal limits for THC and alcohol in 10 European countries; four of these countries have a zero tolerance for THC.

**Table 2:** Legal Δ^9^‐tetrahydrocannabinol (THC) and alcohol limits in Europe

Country	THC levels threshold	Law	Alcohol driving thresholds
Austria	Not fixed. Medications allowed if not impairing	5 Abs 1 StVO	0.05 g/100 ml blood (0.05%)
Belgium	Blood: 1 ng/ml, Saliva: 10 ng/ml	Wegverkeerswet/Loi Circulation Routière 16/March/1968 Art 37, Art 61	0.05 g/100 ml blood (0.05%)
Denmark	Blood: 0.001–0.003 mg/L THC onwards. No sanction if medical justification and aligned levels	Law no. 695of 08‐06‐2017	0.05 g/100 ml blood (0.05%)
Finland	0 level policy. No sanction if a medical justification and aligned levels are the case	Feb 2003, Criminal Code Ch.23, s.3, 4, 8	0.05 g/100 ml blood (0.05%)
France	Any positive tests lead to fine and driving license withdrawal	Feb 3 2003 French law	0.05 g/100 ml blood (0.05%)
Germany	Blood: 1 ng/ml If medication is taken, no fine	24 (2) StVG/1BvR 2652/03, 21/Dec/2014	0.05 g/100 ml blood (0.05%)
Italy	Any positive tests lead to fine and driving license withdrawal	Art 187 Codice della Strada	0.05 g/100 ml blood (0.05%)
The Netherlands	New law with limits expected 2017	Section 8 of the 1994 Road Traffic Act section 1	0.05 g/100 ml blood (0.05%)
Norway	Blood: 0.4 μg/L onwards. No sanction if medical justification	https://helsenorge.no/legemidler/bilkjoring-og-legemidler	0.02 g/100 ml blood (0.02%)
Portugal	Any blood level. Urine: 50 ng/ml leads to blood test	Law no. 18/2007 of 17 May and Portaria 902‐B/2017 of 13 August.	0.05 g/100 ml blood (0.05%)
Spain	Any positive tests lead to fine	RD 1428/2003 21/Nov	0.05 g/100 ml blood (0.05%)
Sweden	0 level policy. No sanction if a medical justification and aligned levels are the case	Law no.1951:649, 1 July 1999, 4 §	0.02 g/100 ml blood (0.02%)
Switzerland	Blood: 1.5 μg/L	VSKV‐ASTRA SR 741.013.1	0.05 g/100 ml blood (0.05%)
United Kingdom	Blood: 2 μg/100 ml	2 March 2015 Traffic law new added list	0.08 g/100 ml blood (0.08%), Scotland 0.05

The knowledge of THC from recreational herbal cannabis and driver impairment is also important in the case of persons being treated with medicinal cannabinoids medications. Extrapolation of epidemiological and experimental studies has often been used to ascertain the role of THC in these situations (Grotenhermen et al., 2007; Ramaekers, Berghaus, van Laar, & Drummer, 2004). Epidemiological studies clearly show an association between more frequent herbal cannabis use, driving within one hour of recreational cannabis smoking, and increased blood THC concentrations with an increased risk of motor vehicle accidents (Hartman & Huestis, 2013). In a case–control study in Australia, drivers with measurable blood THC levels were significantly more likely to be involved in fatal road crashes than drug‐free drivers (OR 2.7), and for drivers with a THC level ≥5 μg/L, the risk was even greater (OR 6.6) (Drummer et al., 2004). Likewise, meta‐analyses of epidemiological studies also confirmed an increased risk of motor vehicle accidents following acute cannabis use (Li et al., 2012; Rogeberg & Elvik, 2016).

Experimental studies have been used to evaluate the effects of THC on cognitive function and driving ability and a review of more recent studies found that drivers under the influence of THC attempt to compensate by driving more slowly, but increasing the complexity of the tasks performed reduced their ability to maintain control (Hartman & Huestis, 2013). Studies involving driving simulators demonstrated that THC resulted in significant impairment of road‐tracking, standard deviation of lateral position (lane weave), and steering wheel variability (Lenné et al., 2010; Ronen et al., 2010).

## 6 THC:CBD

### 6.1 General efficacy and tolerability of THC:CBD oromucosal spray in MS spasticity

THC:CBD oromucosal spray is approved across the EU and in other countries for the management of moderate‐to‐severe MS‐related spasticity in adult patients resistant to other antispasticity medications and who demonstrated clinically significant improvement in spasticity‐related symptoms during an initial one‐month course of therapy. Approval was based on randomized controlled clinical trials (Collin, Davies, Mutiboko, & Ratcliffe, 2007; Collin et al., 2010; Novotna et al., 2011). Observational studies and treatment registries have subsequently confirmed its effectiveness and tolerability in everyday clinical practice. Each 100 μl spray contains 2.7 mg THC and 2.5 mg CBD.

### 6.2 Pivotal clinical trials program for THC:CBD oromucosal spray

The first phase 3 clinical trial findings (Collin et al., 2007, 2010) led the way to an enriched enrollment design study in which only those participants who demonstrated responsiveness to THC:CBD oromucosal spray were eligible for randomization (Novotna et al., 2011). During an initial single‐blind 4‐week trial, 272 (47.6%) of 572 subjects were classified as responders (≥20% numerical rating scale (NRS) improvement), and 241 of these were randomized to double‐blind treatment with THC:CBD oromucosal spray or placebo for 12 weeks. About 75% of the initial responders randomized to THC:CBD oromucosal spray achieved the clinically relevant threshold of a ≥ 30% improvement in NRS vs. baseline; this was significantly higher than the number reaching this threshold in the placebo group (OR 2.73; 95% CI: 1.59, 4.69: *p* = .0003). Clinical improvement in favor of THC:CBD oromucosal spray was also recorded for sleep disturbances (46% improvement vs. placebo) and spasm frequency (51% improvement vs. placebo). These clinical trials supported the regulatory approval of THC:CBD oromucosal spray as a second‐line treatment option for patients with MS spasticity. Regarding tolerability, it should be noted that mild‐to‐moderate transient dizziness or somnolence was experienced by about 10% of patients, and also other less common nonserious adverse events. No special safety concerns, abuse, or dependence cases were raised. In light of these effects, the approved European label states: “*Effects on ability to drive and use machines: Sativex may produce undesirable effects such as dizziness and somnolence which may impair judgement and performance of skilled tasks. Patients should not drive, operate machinery or engage in any hazardous activity if they are experiencing any significant CNS effects such as dizziness or somnolence.”*

### 6.3 Observational studies and registry data for THC:CBD oromucosal spray

Similar effectiveness and tolerability outcomes with THC:CBD oromucosal spray were reported in a retrospective registry analysis (United Kingdom [UK]/Germany/Switzerland) and prospective safety study (Spain) conducted in everyday practice (Etges et al., 2015; Oreja‐Guevara, 2015). In the first 12 months of exposure, approximately two‐thirds of patients in each cohort gained sufficient benefit in their physicians' opinion to warrant continued treatment. The incidence of significant adverse events (AEs) was low, and there was no evidence of addiction, abuse, misuse, or memory impairment. Mean doses of THC:CBD oromucosal spray were lower than those reported in randomized controlled trials (RCTs; ~5–7 vs. >8 sprays/day).

Coinciding with the introduction of THC:CBD oromucosal spray in Italy, a web‐based registry was set up by the health authorities to collect data on all patients prescribed the medicine. Analysis of 1534 patients receiving THC:CBD oromucosal spray from January 2014 to February 2015 (53% female; mean age 50.0 ± 9.6 years) found that 62% were initial responders (≥20% NRS improvement) at month 1 and that 41% of 547 patients who reached the 6‐month evaluation visit were clinically relevant responders (≥30% NRS improvement) (Patti et al., 2016). The mean dose was ~6–7 sprays/day. No new safety signals beyond the approved label for THC:CBD oromucosal spray were observed, and, again, there was no evidence of abuse/misuse.

The prospective, observational MOVE 2 studies from Germany (Flachenecker, Henze, & Zettl, 2014) and Italy (Trojano & Vila, 2015) enrolled similar patient populations (*n* = 335 and 322, respectively) and reported broadly similar outcomes with use of THC:CBD oromucosal spray in everyday clinical practice. The main difference between studies was the higher proportion of initial responders to THC:CBD oromucosal spray at month 1 in Italy compared with Germany (82.8 vs. 41.7%), which may reflect some positive bias toward a new intervention in Italy, as clinically relevant responder rates at month 3 were more aligned. THC:CBD oromucosal spray was tolerated equally well in the two studies. <20% of patients reported AEs, which consisted mainly of mild‐to‐moderate transient episodes of somnolence or dizziness. Mean daily doses were lower than in RCTs.

### 6.4 THC:CBD pharmacokinetics

Determining the pharmacokinetic (PK) properties of THC following administration of THC:CBD oromucosal spray is important to help us better understand whether the product may affect cognitive function and driving ability.

**Figure 1:**
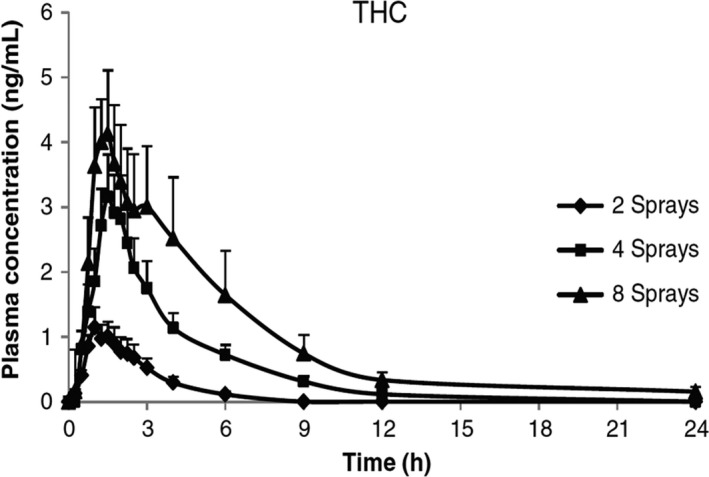
Mean (+SEM) THC plasma concentration curve over time following administration of THC:CBD (two, four, and eight sprays equivalent to 5.4, 10.8, and 21.6 mg THC) (Stott et al., 2013)

To investigate the PK of THC:CBD oromucosal spray, 24 healthy male subjects were divided into three groups and received single doses of multiple consecutive sprays: two sprays (5.4 mg THC, *n* = 6); four sprays (10.8 mg THC, *n* = 12); and eight sprays (21.6 mg THC, *n* = 6) (Figure 1) (Stott, White, Wright, Wilbraham, & Guy, 2013). It should be noted that THC:CBD oromucosal spray administration in daily practice, according to the approved label, states that the required sprays (usually 6–7/day on average) should be spread throughout the day according to individual response and tolerability and that there should be 15 min between individual sprays. This was followed by a multiple‐dose study in which the same dosages were taken once daily for nine days. The main THC PK parameters are summarized in Table 3 (Stott et al., 2013). In a second study, equivalent doses of THC (approximately 5 mg and 15 mg) administered as THC:CBD oromucosal spray or as oral synthetic THC resulted in similar PK profiles with no clinically relevant differences between them (Karschner, Darwin, Goodwin, Wright, & Huestis, 2011). This indicates that CBD does not materially impact the PK of THC and, therefore, PK changes do not explain its modulation of THCs psychological effects (Karschner et al., 2011). Importantly, in both of these studies, the THC plasma levels were magnitudes lower than levels reported after smoking herbal cannabis (Huestis & Cone, 2004; Huestis, Henningfield, & Cone, 1992). For example, smoking cannabis with a standardized THC content of 33.8 mg resulted in a C_max_ of 162.2 μg/L and t_max_ of 9 min (Huestis & Cone, 2004), while with THC:CBD oromucosal spray, a C_max_ of 5.4 μg/L and t_max_ of 60 min were observed after a dose of eight sprays in a row (21.6 mg THC) (Table 4). Significantly lower C_max_ values were recorded after lower dosages of THC:CBD oromucosal sprays, while t_max_ was between 60 and 90 min (Table 3). The markedly slower delivery and reduced exposure of THC following oromucosal delivery are reassuring and almost certainly explain the very low propensity for psychological effects / abuse.

**Table 3:** Summary of mean (standard deviation) pharmacokinetic parameters in healthy male subjects administered three different doses of THC:CBD oromucosal spray (Stott et al., 2013)

Parameter	Δ^9^‐tetrahydrocannabinol (THC) number of sprays (dose)		
	Two sprays (5.4 mg)	Four sprays (10.8 mg)	Eight sprays (21.6 mg)
Single‐dose study			
C_max_ (μg/L)	1.5 (0.5)	4.0 (2.3)	5.4 (2.4)
T_max_ (h)	1.0 (0.8–1.5)	1.5 (0.8–2.0)	1.0 (0.8–1.5)
AUC_0‐inf_ (μg/L h)	3.5 (1.8)	12.5 (7.3)	24.7 (20.7)
Multiple‐dose study			
C_max_ (μg/L)	1.4 (0.6)	2.7 (1.5)	6.9 (2.1)
C_min_ (μg/L)	NC	0.1 (0.03)	0.4 (0.2)
T_max_ (h)	1.6 (range 1.0–4.0)	1.5 (range (0.8–2.5)	3.3 (1.0–6.0)
AUC_0‐τ_ (μg/L h)	4.1 (1.6)	9.9 (3.7)	39.9 (4.7)

AUC_0‐inf_, area under the plasma concentration versus time curve from time 0 to infinity; AUC_0‐τ_, area under the plasma concentration versus time curve over the final dosing interval (0‐24 h); C_max_, maximum plasma concentration; C_min_, minimum plasma concentration; NC, not calculated; T_max_, time to C_max_.

**Table 4:** Pharmacokinetic parameters for THC:CBD oromucosal spray (Sativex), for vaporized THC extract and smoked cannabis (Huestis et al., 1992; Summary of Product Characteristics, Sativex Oromucosal Spray, 2017)

	THC pharmacokinetic parameters		
	C_max_ ng/ml	T_max_ min	AUC _(0‐t)_ ng/ml min
Sativex (providing 21.6 mg THC)	5.4	60	1,362
Inhaled vaporized THC extract (providing 8 mg THC)	118.6	17	5,987.9
Smoked cannabis^a^ (providing 33.8 mg THC)	162.2	9	No data

AUC_0‐t_, area under the plasma concentration versus time curve (0‐designated time); C_max_, maximum plasma concentration; T_max_, time to C_max_.

a Huestis et al., Journal of Analytical Toxicology 1992; 16:276–82.

### 6.5 THC:CBD oromucosal spray and driving: real‐world data/pharmacovigilance

**Figure 2:**
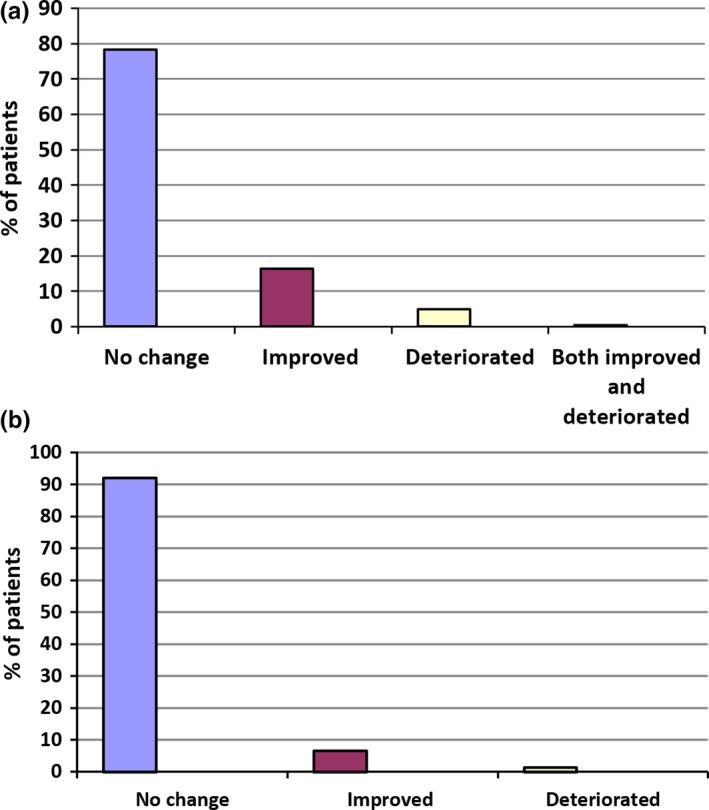
Change in driving ability in patients with MS spasticity treated with THC:CBD and who were able to drive when included onto an observational product registry in Germany, Switzerland, and the UK (a) (Etges et al., 2016), or an observational study in Spain (b) (Oreja‐Guevara, 2015). (a) Product registry (Germany, Switzerland, and the UK;* n* = 387). (b) Observational study data, Spain (*n* = 77)

A postmarketing risk management registry opened in the UK in 2010, followed by Germany in 2012, and was extended to include Switzerland in 2015 (Etges et al., 2016). The registry is a noninterventional safety study for patients prescribed THC:CBD oromucosal spray, with the objective of monitoring the emergence of new safety signals that may not be apparent in shorter RCTs, where patients have had to meet stringent eligibility criteria. Prescribers were requested to answer targeted questions related to areas of special interest identified in a risk management plan concerning mainly neurological and psychiatric disorders, but also monitored changes in driving ability. In early 2015, the UK arm of the registry closed after the authorities concluded that sufficient data had been obtained from the UK patients to characterize the safety profile of long‐term treatment with THC:CBD oromucosal spray. Limited data continue to be collected under national approval requirements in Germany and Switzerland. The global postmarketing safety exposure for THC:CBD oromucosal spray is now estimated to be above 55,000 patient‐years, and no driving impairment safety risk has emerged. Pertinent to this review, of the 941 patients entered onto the registry, 387 had data regarding driving ability and 303 of these reported no change in driving ability (Figure 2a). Of the remaining 84 patients, the majority (*n* = 63) reported an improvement in driving ability and only 19 patients noted that their ability to drive had deteriorated. The authors speculated that the improvement in driving ability may be related to less spasticity and/or better cognitive function (Etges et al., 2016).

In another long‐term prospective observational study that was performed in Spain, driving ability was included as a possible AE of special interest in patients with moderate‐to‐severe resistant MS‐related spasticity treated with THC:CBD oromucosal spray (Oreja‐Guevara, 2015). Safety evaluations were performed after 6 and 12 months' exposure to THC:CBD oromucosal spray. During the course of this study, 93% of patients rated their driving ability as not changed/not reported/not relevant, while 5% rated it as improved, and 1% as deteriorated (Figure 2b).

The specific impact of THC:CBD oromucosal spray treatment on driving ability was investigated in a formal ad hoc observational study in patients with moderate‐to‐severe resistant MS spasticity in Germany (Freidel et al., 2015). Thirty‐three patients with MS spasticity about to start treatment with add‐on THC:CBD oromucosal spray performed a set of five driving set procedures from a validated computerized test battery at baseline, and the tests were repeated after 4–6 weeks' treatment. The tests measured different dimensions associated with driving such as reaction speed, concentration, orientation, stress tolerance, and attention. Compared with baseline, there was no significant reduction in test results in patients treated with THC:CBD oromucosal spray. Indeed, the majority of tests showed a slight improvement, including the overall driving test result, and in the case of “determination,” the result was significantly improved at the end of treatment (*p* = .026). Patient‐assessed MS spasticity scores were significantly improved during the course of this study (*p* < .0001).

## 7 DISCUSSION

Assessment of driving ability in patients with neurological disorders is an important consideration as cessation of driving can have a profound impact on the individual's QoL. Loss of mobility often equates to loss of independence, poorer relations with family and friends, and reduced social activity (Rapport et al., 2006). In individuals with MS, research has shown that driving performance may be negatively affected by the disease. For example, recent studies show that both cognitive problems and spasticity (muscle stiffness or spasms) affect driving performance, putting the person at an increased risk of a motor vehicle accident (Niewoehner & Thomas, 2017). Another important consideration is that people with MS may be taking several medications to manage their MS and its associated symptoms (spasticity, mood changes, bladder problems, walking problems, and/or pain). It is imperative that the effects of these treatments on factors that could influence driving performance, such as wakefulness, concentration, coordination, and reaction times, are clearly understood.

In this article, we review the evidence for THC:CBD oromucosal spray which is approved across Europe as second‐line therapy for patients with moderate‐to‐severe MS‐related spasticity resistant to first‐line agents such as baclofen (Summary of Product Characteristics, Sativex Oromucosal Spray, 2017). Patients being treated with THC:CBD oromucosal spray have multiple factors that could affect their driving ability: the disease; spasticity treatments; concomitant medications administered for the disease or MS symptoms, and the effects of the drug per se.

In terms of efficacy, a number of pivotal RCTs and large observational studies have clearly shown the benefits of THC:CBD oromucosal spray in patients who respond to an initial trial of therapy (Flachenecker, 2016). The same studies have generally highlighted THC:CBD oromucosal spray is tolerated well. However, THC:CBD oromucosal spray is associated with mild‐to‐moderate transient drowsiness and somnolence on some occasions, and, if affected, such patients should be warned not to drive while these adverse events are present (Summary of Product Characteristics, Sativex Oromucosal Spray, 2017). The results from the studies and registries reviewed herein did not find any evidence of an increase in motor vehicle accidents associated with THC:CBD oromucosal spray in patients with MS. Furthermore, a majority of patients reported an improvement in driving ability after starting treatment with THC:CBD oromucosal spray, and the authors speculated that this may be related to less spasticity and/or better cognitive function (Etges et al., 2016).

The risk to people treated with THC:CBD oromucosal spray of failing a roadside drugs' test for THC is an issue which is not so easy to respond to. Based on PK data, the answer is dose‐ (number of sprays) and time‐related. The threshold level in the majority of European countries is approximately 1–2 μg/L (Table 2). After nine consecutive days of administration of two sprays of THC:CBD oromucosal spray (containing 5.4 mg THC), the C_max_ of THC was 1.4 μg/L and occurred approximately 1 hr after administration (Etges et al., 2015). So, at this double the standard single spray administration level, persons using THC:CBD oromucosal spray would likely fail a 1 μg/ml THC threshold roadside drugs test for a certain period following administration. However, interpersonal variability might be relevant.

Impairment is used in some jurisdictions to determine the individual's ability to drive. However, based on the current literature, THC blood levels associated with impairment are not entirely clear (Asbridge et al., 2012; Kelly et al., 2004; Wong et al., 2014). Again, this is probably as a result of intersubject variability. In addition, it has been suggested that persons using compounds that impair risk may try to overcome such impairment through compensating behaviors such as driving more slowly and avoiding high‐risk situations (Kelly et al., 2004). These are all considerations for physicians treating patients with MS and who may need to make a decision regarding driving suitability. While physicians were generally good at making such decisions, a small proportion of patients with MS (not on THC:CBD oromucosal spray) who were allowed to drive failed an on‐road assessment, mainly for visual reasons (Ranchet, Akinwuntan, Tant, Neal, & Devos, 2015). Even though THC:CBD oromucosal spray can result in dose‐related levels of THC above the legal threshold for a number of countries, there is no evidence that it is associated with further impairment of driving performance in patients with MS based on a significant amount of postmarketing safety data available. This may be related to the slower delivery of the drug observed in PK studies.

## 8 CONCLUSIONS

MS is a chronic neurological disease which is associated with a spectrum of symptoms that may impact the functional status, overall well‐being, and QoL of the individual. The disease and its treatments have the potential to impair driving ability, an ability that most affected patients will want to retain as long as possible. The cannabinoid‐based medication THC:CBD oromucosal spray can be successfully prescribed to patients with MS to help relieve spasticity and associated symptoms. It has not been shown to impair driving if adverse events such as somnolence and dizziness are not present. However, periodic assessment of the MS patient's driving ability is recommended, especially after relapses and treatment changes. Blood THC measurements might record levels above the authorized thresholds for some countries at different time points after intake. Recommendations regarding driving after administration of THC:CBD oromucosal spray need to be considered on a country‐by‐country basis, to adapt to specific laws. The EU label states that patients should not drive, operate machinery, or engage in any hazardous activity if they are experiencing any significant CNS effects such as dizziness or somnolence. THC blood levels after administration of THC:CBD oromucosal spray will be under the allowed threshold a few hours after intake in most cases (except when the threshold is “zero”). However, individual variability does not guarantee this. Having a medical certificate confirming the medical prescription of THC containing treatment and the absence of driving impairment while on the medication is therefore recommended. The traffic authorities of countries where the medical prescription of a THC containing medicine is permitted and, as long as physician reports lack of impairment in driving ability, should be asked to make specific considerations for such individuals in their regulations. This is currently the case in Austria, Denmark, Finland, Germany, and Sweden.

## CONFLICT OF INTEREST

EGC has received personal compensation for serving on scientific advisory boards for Almirall, Biogen, Merck, Novartis, Genzyme, and Teva and has received speaker honoraria from Biogen, Genzyme, Novartis, Merck, and Teva, and her department has received unrestricted research grants from Novartis and Genzyme.
